# Erythrophagocytosis of Lead-Exposed Erythrocytes by Renal Tubular Cells: Possible Role in Lead-Induced Nephrotoxicity

**DOI:** 10.1289/ehp.1408094

**Published:** 2014-10-10

**Authors:** So-Youn Kwon, Ok-Nam Bae, Ji-Yoon Noh, Keunyoung Kim, Seojin Kang, Young-Jun Shin, Kyung-Min Lim, Jin-Ho Chung

**Affiliations:** 1 College of Pharmacy, Seoul National University, Seoul, Korea; 2 College of Pharmacy, Hanyang University, Ansan, Korea; 3 College of Pharmacy, Ewha Womans University, Seoul, Korea; * These authors contributed equally to this research.

## Abstract

Background: Nephrotoxicity associated with lead poisoning has been frequently reported in epidemiological studies, but the underlying mechanisms have not been fully described.

Objectives: We examined the role of erythrocytes, one of the major lead reservoirs, in lead-associated nephrotoxicity.

Methods and results: Co-incubation of lead-exposed human erythrocytes with HK-2 human renal proximal tubular cells resulted in renal tubular cytotoxicity, suggesting a role of erythrocytes in lead-induced nephrotoxicity. Morphological and flow cytometric analyses revealed that HK-2 cells actively phagocytized lead-exposed erythrocytes, which was associated with phosphatidylserine (PS) externalization on the erythrocyte membrane and generation of PS-bearing microvesicles. Increased oxidative stress and up-regulation of nephrotoxic biomarkers, such as NGAL, were observed in HK-2 cells undergoing erythrophagocytosis. Moreover, TGF-β, a marker of fibrosis, was also significantly up-regulated. We examined the significance of erythrophagocytosis in lead-induced nephrotoxicity in rats exposed to lead via drinking water for 12 weeks. We observed iron deposition and generation of oxidative stress in renal tissues of lead-exposed rats, as well as the histopathological alterations such as tubulointerstitial lesions, fibrosis, and up-regulation of KIM-1, NGAL, and TGF-β.

Conclusions: Our data strongly suggest that erythrophagocytosis and subsequent iron deposition in renal tubular cells could significantly enhance nephrotoxicity following lead exposure, providing insight on lead-associated kidney damages.

Citation: Kwon SY, Bae ON, Noh JY, Kim K, Kang S, Shin YJ, Lim KM, Chung JH. 2015. Erythrophagocytosis of lead-exposed erythrocytes by renal tubular cells: possible role in lead-induced nephrotoxicity. Environ Health Perspect 123:120–127; http://dx.doi.org/10.1289/ehp.1408094

## Introduction

Although environmental lead contamination has declined significantly since the 1970s, lead exposure is still observed in children and industrial workers, and even in the general population (Hernberg 2000). The average adult blood lead level (BLL) is 1–2 μg/dL, and the U.S. Centers for Disease Control and Prevention (CDC) defines lead poisoning as a BLL > 10 μg/dL (0.5 μM) (CDC 1997). Epidemiological and toxicological studies have reported lead-induced toxicity in the nervous, cardiovascular, and renal systems [Agency for Toxic Substances and Disease Registry (ATSDR) 2007]. The association between lead exposure and nephrotoxicity has been well-established, even in a population with BLLs as low as 5 μg/dL (Ekong et al. 2006). Damage in kidney function is associated with albuminuria, reduced glomerular filtration rate, and decreased creatinine clearance in lead-exposed populations (Fadrowski et al. 2010; Navas-Acien et al. 2009). Histopathologically, renal impairment associated with lead poisoning is characterized by proximal tubular nephropathy, glomerular sclerosis, and fibrosis in peritubular and interstitial lesions (Cramér et al. 1974; Diamond 2005; Goyer 1989; Loghman-Adham 1997).

Oxidative stress has been suggested to be the most convincing mechanism underlying lead-associated nephrotoxicity (Daggett et al. 1998; Wang et al. 2009). Pro-oxidant and antioxidant balance, along with decreased glutathione and increased lipid peroxidation, occurs in the kidney following lead exposure in animal models (Daggett et al. 1998; Liu et al. 2012b; Patra et al. 2001; Wang et al. 2010). There have been several attempts to determine how lead increases oxidative stress in the kidney (Stacchiotti et al. 2009; Wang et al. 2009, 2011), but the exact mechanism(s) has not been clearly elucidated.

There is increasing evidence (Madsen et al. 1982; Mimura et al. 2008; Sheerin et al. 1999; Trump et al. 1969) that the kidney may play a role in the clearance of erythrocytes. Infiltration of erythrocytes has been observed in proximal tubules and tubular lumen of renal biopsies from patients with acute glomerulonephritis and hematuria (Trump et al. 1969) as well as in those from patients with acute renal failure (Mimura et al. 2008). Iron deposition in the kidney was also found in patients with various renal diseases (Wang et al. 2001), suggesting that the retention of iron-rich erythrocytes in the kidney may play a role in the pathogenesis of kidney diseases. Proximal tubular epithelial cells are capable of phagocytizing and degrading erythrocytes (Madsen et al. 1982; Sheerin et al. 1999), a phenomenon known as erythrophagocytosis. Erythrophagocytosis, which is primarily carried out by macrophages in the spleen and liver (Knutson and Wessling-Resnick 2003; Otogawa et al. 2007), occurs when aged or damaged erythrocytes are phagocytized and cleared from systemic circulation. This process is mediated by externalized phosphatidylserine (PS) on the outer membrane (Kobayashi et al. 2007; Mercer and Helenius 2008) and by PS-bearing microvesicles (MVs) (Knutson and Wessling-Resnick 2003; Otogawa et al. 2007). Proximal tubule cells have been reported to actively phagocytize erythrocytes in renal injury (Madsen et al. 1982; Sheerin et al. 1999), but the toxicological significance of this process in the etiology of heavy metal–associated renal diseases remains to be established.

More than 99% of blood lead accumulates in erythrocytes, suggesting that erythrocytes may be a major target of systemic lead poisoning (Hernández-Avila et al. 1998; Schütz et al. 1996). We recently demonstrated that lead significantly increased PS externalization in erythrocytes and enhanced erythrophagocytosis by macrophages in the spleen (Jang et al. 2011). Fowler et al. (1980) observed iron deposition in renal proximal tubule cells, along with lead-associated histopathological lesions in the kidney, following administration of lead via drinking water to rats. In the present study we examined the role of erythrocytes in lead-induced nephrotoxicity *in vitro* in a co-culture system as well as *in vivo* in rats. On the basis of available evidence, we hypothesized that lead-induced PS externalization in erythrocytes promotes erythrophagocytosis by renal tubular cells, contributing to lead-associated kidney damage.

## Materials and Methods

*Chemicals*. We obtained lead(II) acetate (Pb^2+^), calcium chloride, glutaraldehyde solution, osmium tetroxide, bovine serum albumin (BSA), HEPES, sodium citrate, dimethyl sulfoxide (DMSO), isopropanol, 3-(4,5-dimethylthiazol-2yl)-2,5-diphenyl-2H-tetrazolium bromide (MTT), and dihyroethidium (DHE) from Sigma Chemical Co. (St. Louis, MO, USA). Phycoerythrin (PE)-labeled monoclonal antibody against human glycophorin A (anti-glycophorin A-RPE) was from Dako (Glostrub, Denmark), and fluo-4 acetoxymethyl ester (Fluo-4 AM) was from Molecular Probes (Eugene, OR, USA). We obtained fluorescein isothiocyanate (FITC)-labeled annexin V (annexin V-FITC) from Pharmingen (San Diego, CA, USA) and 5-(-6)-carboxyfluorescein diacetate (CFDA), calcein red-orange, anti-CD13-PE, anti-CD13-perCP-Cy5.5, and H2-DCFDA from Invitrogen (Eugene, OR, USA). Keratinocyte serum-free media kit was from Gibco BRL Life Technologies Inc. (Grand Island, NY, USA). All other reagents were of the highest purity available.

### *In Vitro* Experiments

*Preparation of erythrocytes*. After obtaining approval from the Ethics Committee of Health Service Center at Seoul National University, we obtained human blood from healthy Korean male donors who provided informed consent (*n* = 20; 20–29 years of age). Blood was collected using a vacutainer containing acid citrate dextrose via a 21-gauge needle (Becton Dickinson, Franklin Lakes, NJ, USA) on the day of the experiment. Platelet-rich plasma and buffy coat were removed by aspiration after centrifugation at 300 × *g* for 15 min. Packed erythrocytes were washed three times with sterilized phosphate-buffered saline (PBS: 1 mM KH_2_PO_4_, 154 mM NaCl, 3 mM Na_2_HPO_4_, pH 7.4) and once with filter-sterilized Ringer’s solution (125 mM NaCl, 5 mM KCl, 1 mM MgSO_4_, 32 mM HEPES, 5 mM glucose, pH 7.4). Washed erythrocytes were resuspended in Ringer’s solution to a cell concentration of 5 × 10^7^ cells/mL. CaCl_2_ (final concentration, 1 mM) was added to erythrocytes prior to use. The erythrocytes were used immediately after isolation without storage.

*Cell culture*. We used renal proximal tubular cells, the cell population most susceptible to xenobiotic-induced toxicity in the kidney. Human proximal tubular epithelial cells [HK-2; ATCC (American Type Culture Collection), Manassas, VA, USA] were maintained in keratinocyte serum-free media supplemented with recombinant epidermal growth factor, bovine pituitary extract (BPE), and 1% penicillin/streptomycin at 37°C under a 5% CO_2_ atmosphere (Ryan et al. 1994).

*Cell viability measurement*. Cell viability was measured using the MTT assay as previously described (Thiébault et al. 2007) with slight modification. To evaluate the effect of Pb^2+^ on cell viability, we incubated HK-2 cells (2 × 10^5^ cells/well in 6-well plates) with either vehicle (1% distilled water) or Pb^2+^ (10 or 20 μM) for 24 hr. Cells were then incubated with MTT (0.5 mg/mL) for 2 hr and washed. The converted formazan was dissolved in 100% DMSO, and the absorbance at 570 nm was measured in a SpectraMax spectrophotometer (Molecular Devices, Sunnyvale, CA, USA).

In experiments to examine the contribution of erythrocytes, erythrocytes were incubated with vehicle or Pb^2+^ (10 or 20 μM) for 1 hr and then removed by centrifugation. HK-2 cells were co-incubated with Pb^2+^-treated erythrocytes (1 × 10^7^ cells/well) for 24 hr. After co-incubation, erythrocytes were removed and MTT was added as described above.

*Morphological examination*. Erythrocytes were treated with either vehicle or Pb^2+^ (20 μM) for 1 hr and washed. HK-2 cells were then co-incubated with the vehicle- or Pb^2+^-treated erythrocytes for 3 hr. Morphology was examined in order to measure the interaction between erythrocytes and HK-2 cells using phase-contrast microscopy (Olympus IX70; Olympus Corporation, Tokyo, Japan). For scanning electron microscopic observation (Jang et al. 2011; Noh et al. 2010), HK-2 cells co-incubated with vehicle- or Pb^2+^-treated erythrocytes were fixed with 2% glutaraldehyde for 1 hr at 4°C. Cells were washed three times with PBS and treated with 1% osmium tetroxide for 30 min at room temperature. After washing with PBS, the samples were dehydrated serially in 50%, 70%, 90%, and 100% ethanol. After drying and coating with gold, the images were obtained on scanning electron microscope (JEOL, Tokyo, Japan).

*Measurement of* in vitro *erythrophagocytosis*. After treatment with vehicle or Pb^2+^ (10 or 20 μM) for 1 hr, erythrocytes were treated with 10 μM CFDA for 30 min. HK-2 cells were then co-incubated with vehicle- or Pb^2+^-treated erythrocytes for 3 hr. After co-incubation, HK-2 cells were harvested and washed several times to remove remnant erythrocytes. Anti-CD13-PE was used to identify HK-2 cells. Samples were analyzed on a FACScalibur flow cytometer equipped with an argon ion laser emitting at 488 nm (Becton Dickinson). Cells were identified based on their forward and side scatter (FSC and SSC, respectively) characteristics. The fluorescence signals from FL1 and FL2 were also analyzed. We collected and analyzed data from 10,000 events positive for PE (FL1) using CellQuest Pro software (Becton Dickinson). Cells with double positive signals for PE (FL1) and CFDA (FL2) were counted as HK-2 cells with erythrophagocytosis.

To investigate the role of externalized PS in erythrophagocytosis, we used purified annexin V (0.5 μM) that binds to the externalized PS and interrupts PS-mediated phenomena. Erythrocytes were incubated with purified annexin V for 2 min and then exposed to lead for 1 hr. Erythrophagocytosis was measured as described above.

*Analysis of PS exposure and MV generation*. Erythrocytes were treated with vehicle or Pb^2+^ (10 or 20 μM) for 1 hr at 37°C, and then incubated with annexin V-FITC, anti-glycophorin A antibody, and CaCl_2_ (2.5 mM) for 30 min at room temperature in the dark to detect PS externalization. PE-labeled anti-glycophorin A was used to identify erythrocytes and erythrocyte-derived MVs. Isotype controls for measurement of nonspecific annexin V binding were stained with annexin V-FITC in the presence of EDTA (2.5 mM) instead of CaCl_2_. Samples were analyzed on the flow cytometer as described above.

*Measurement of reactive oxygen species (ROS)* in vitro. Erythrocytes treated with vehicle or Pb^2+^ (10 or 20 μM) for 1 hr were then loaded with 10 μM of calcein red-orange for 30 min. The HK-2 cells were loaded with H_2_DCFDA for 30 min and then co-incubated with vehicle- or Pb^2+^-treated erythrocytes for 4 hr. After co-incubation, HK-2 cells were harvested and washed several times to remove excess erythrocytes. Anti-CD13-perCP-Cy5.5 was used to identify HK-2 cells. Samples were analyzed by flow cytometry. As described above, HK-2 cells with erythrophagocytosis were defined as cells with double positive signals for perCP-Cy5.5 (FL3) and calcein red-orange (FL1). ROS generation was analyzed using the dichlorofluorescein signal from the cells with erythrophagocytosis.

In vitro *quantitative real-time polymerase chain reaction (PCR)*. After co-incubation of HK-2 cells with vehicle- or Pb^2+^-treated erythrocytes for 4 hr, total mRNA was isolated from HK-2 cells using the Easy-Blue Total RNA Extraction Kit (Intron Biotechnology, Seongnam, Korea). Isolated mRNA was quantified using a Nanodrop spectrophotometer (Thermo Scientific, Wilmington, DE, USA), and cDNA was synthesized from isolated RNA using the iScript™ cDNA synthesis kit (Bio-Rad, Hercules, CA, USA). We used quantitative real-time PCR (qRT-PCR) to determine the mRNA levels for NGAL (neutrophil gelatinase-associated lipocalin) and KIM-1 (kidney injury molecule-1), both or which are representative biomarkers of nephrotoxicity, and TGF-β (transforming growth factor β), an important mediator of renal fibrosis. qRT-PCR was conducted using 2× SYBR green reaction buffer mixed with 0.5 μg cDNA and forward/reverse primers. Quantification of gene copies was carried out on a CFX96™ Real-Time PCR Detection System using iQ™ SYBR Green supermix (both from Bio-Rad). PCR cycles consisted of an initial step at 95°C for 3 min followed by 45 cycles at 95°C for 10 sec, 55°C for 30 sec, and 72°C for 10 sec. Relative mRNA expressions were calculated by the comparative CT method (2^–ΔΔCt^) and normalized to the endogenous *18S* control. The specific primer sequences were as follows: *h18S*, forward: GTA ACC CGT TGA ACC CCA TT, reverse: CCA TCC AAT CGG TAG TAG CG; *hKIM1*, forward: GAA CAT AGT CTA CTG ACG GCC AAT AC, reverse: GAA CCT CCT TTT TGA AGA AAT ACT TTT T; *hLCN2* (NGAL), forward: TCA CCT CCG TCC TGT TTA GG, reverse: CGA AGT CAG CTC CTT GGT TC; *hTGFB1*, forward: CCC AGC ATC TGC AAA GCT C, reverse: GTC AAT GTA CAG CTG CCG CA.

### *In Vivo* Experiments

*Animal treatment*. All animal protocols were approved by the Ethics Committee of the Animal Service Centre at Seoul National University, and the animals were treated humanely and with regard for alleviation of suffering. Male Sprague-Dawley rats (Samtako Co., Korea) weighing 200–250 g were used in all experiments. Before the experiments, animals were acclimated for 1 week in the laboratory animal facility, maintained at constant temperature (22 ± 2°C) and humidity (55 ± 5%) with a 12-hr light/dark cycle. Three rats were housed per cage (width, 260 mm; depth, 420 mm; height, 180 mm). Food (Purina Mills, Seongnam, Korea) and water were provided *ad libitum*. Lead was not assessed in food or untreated water, but BLLs in untreated rats were below the detection limit (0.5 ppb). We conducted our *in vivo* experiments in two independent trials, with rats randomly assigned to treatment groups. In the first trial, rats (*n* = 4/treatment group) were treated with 0 ppm or 1,000 ppm Pb^2+^ in drinking water for 12 weeks. In the second trial, rats were treated with Pb^2+^ at 0, 250, or 1,000 ppm (*n* = 5, 6, and 5, respectively) in drinking water for 12 weeks, and total mRNA was isolated from rat kidneys. The purity of one mRNA sample from a 250-ppm lead-treated rat was not good enough for qRT-PCR, thus leaving 5 rats/treatment group. We observed no significant difference in body weight between the groups. The rats were euthanized at various times during the day, in a random sequence, by exsanguination from the abdominal aorta under ether anesthesia. Blood, spleen, and kidney samples were collected and processed for biochemical analysis. Each isolated kidney was blotted carefully, weighed, and immediately fixed or frozen for further histological examination, isolation of mRNA, or quantification of lead level. Spleens were fixed in 10% formalin for histological examination. Whole blood was used for isolation of serum or was immediately frozen for quantification of lead level. Serum, prepared by centrifugation of blood, was used to measure blood urea nitrogen (BUN) by the enzymatic-kinetics method (Neodin Vetlab, Seoul, Korea). Lead levels in frozen blood and kidney were analyzed by inductively coupled plasma mass spectrometry by the National Center for Inter-University Research Facilities, Seoul National University (Seoul, Korea).

*qRT-PCR analysis of kidney.* Total mRNA was prepared from kidney samples isolated from rats treated with Pb^2+^ (0, 250, or 1,000 ppm). Frozen kidney tissue was homogenized in TRIzol reagent (Life Technologies, Carlsbad, CA), and mRNA was further isolated with chloroform and isopropanol. mRNA conversion to cDNA and qRT-PCR were conducted as described above. Relative mRNA expression of *LCN2* (*NGAL*), *KIM1*, and *TGFB1* were calculated by the comparative CT method (2^–ΔΔCt^), normalized to the endogenous *18S* control. The specific primer sequences were as follows: *r18S*, forward: GTA ACC CGT TGA ACC CCA TT, reverse: CCA TCC AAT CGG TAG TAG CG; *rKIM1*, forward: GAA CAT AGT CTA CTG ACG GCC AAT AC, reverse: GAA CCT CCT TTT TGA AGA AAT ACT TTT T; *rLCN2*, forward: TCA CCT CCG TCC TGT TTA GG, reverse: CGA AGT CAG CTC CTT GGT TC; *rTGFB1*, forward: GGA CTA CTA CGC CAA AGA AG, reverse: TCA AAA GAC AGC CAC TCA GG.

*Histological examination*. To assess histological damage, iron accumulation, ROS generation, and collagen accumulation, we examined sections of kidney tissues using staining with hematoxylin and eosin (H&E), Prussian blue, DHE, and Masson trichrome, respectively. Formalin-fixed kidney tissues were used except for DHE staining, in which optimum cutting temperature (OCT)-fixed frozen tissues were used. Kidneys were fixed with 10% formalin, and tissue specimens were cut into 4-μm thick sections and stained with H&E, Prussian blue, or Masson trichrome by Reference Biolabs (Seoul, Korea). Formalin-fixed spleen tissues were handled as described for kidneys, with Prussian blue staining used to measure iron accumulation. Slides were viewed using a bright field microscope (Olympus CX41). Histopathological alterations were identified as focal nephropathy, glomerulonephropathy, or tubulointerstitial lesions by Byung-il Yoon (College of Veterinary Medicine, Kangwon National University, Chuncheon, Korea). For ROS determination, kidneys were frozen in OCT compound, cut into 4-μm thick sections, and incubated in DHE solution in the dark for 30 min. Sections were then washed with PBS and examined under fluorescence microscopes (Carl Zeiss Axiovert 200M; Carl Zeiss Microscopy, Oberkochen, Germany).

*Statistical analysis*. Means ± SEs were calculated for all treatment groups. Data were subjected to one-way analysis of variance (ANOVA) followed by Duncan’s multiple range test or Student’s *t*-test to determine the statistical significance. In all cases, a *p-*value of < 0.05 was considered significant.

## Results

**Figure 1:**
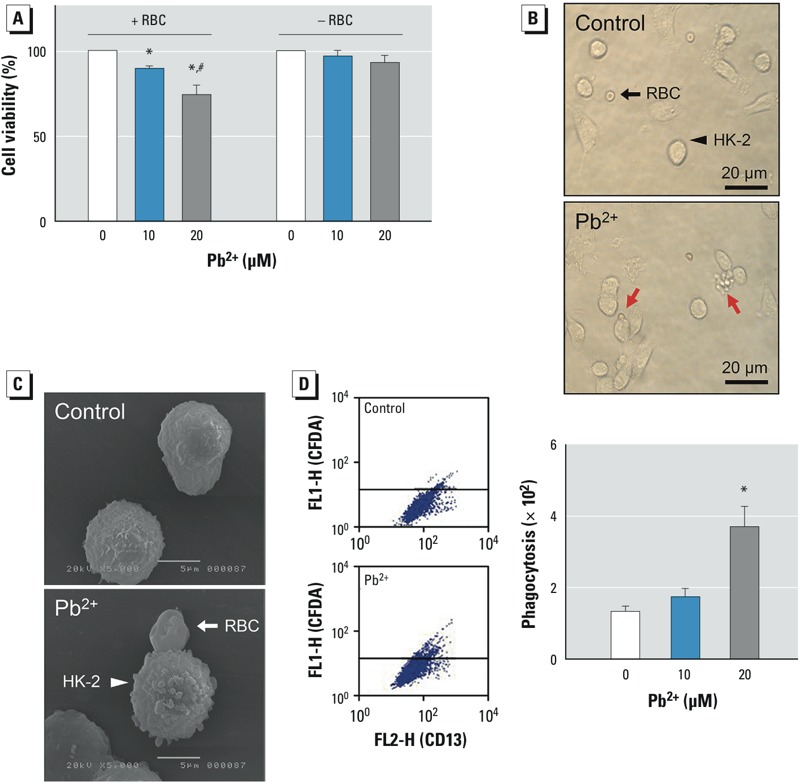
Effects of Pb^2+^-treated human erythrocytes [red blood cells (RBCs); 0 (control), 10, or 20 μM Pb^2+^] on HK-2 renal tubular epithelial cells. (*A*) Cell viability measured in HK-2 cells with (+) or without (–) co-incubation with Pb^2+^-treated erythrocytes. (*B*) Interactions were observed between Pb^2+^-treated erythrocytes and HK-2 cells (bottom; red arrows) but not between control erythrocytes and HK-2 cells (top; black arrows); bars = 20 μm. (*C*) Adherence of control (top) and Pb^2+^-treated (bottom) erythrocytes to HK-2 cells examined using scanning electron microscopy; bars = 5 μm. (*D*) Representative histograms (left) and the numbers of HK-2 cells that were fluorescence-positive by engulfment of CFDA-loaded erythrocytes (right) as analyzed by flow cytometry. Values shown are the mean ± SE of more than three independent experiments. *p < 0.05 compared with the corresponding control, determined by one-way ANOVA followed by Duncan’s multiple range test. ^#^*p *< 0.05 compared with HK-2 cells treated with Pb^2+^ 20 μM in the absence of erythrocytes, determined by Student’s *t*-test.

*Pb^2+^-induced erythrophagocytosis by renal tubular epithelial cells*. To examine the role of erythrocytes in lead-associated kidney injury, human erythrocytes treated with Pb^2+^ (0, 10, or 20 μM) for 1 hr were co-incubated with HK-2 cells, as described in “Materials and Methods.” As shown in Figure 1A, co-incubation of Pb^2+^-treated erythrocytes with HK-2 cells for 24 hr significantly reduced HK-2 cell viability, whereas Pb^2+^ treatment alone (with no erythrocytes added) failed to affect HK-2 viability, supporting a central role of erythrocytes in Pb^2+^-induced HK-2 cytotoxicity. In microscopic examination, we observed adherence of Pb^2+^-treated erythrocytes to HK-2, whereas untreated discocytic erythrocytes did not bind to HK-2 cells (Figure 1B). Images from scanning electron microscopy further confirmed the interaction between HK-2 cells and Pb^2+^-treated spherocytic erythrocytes, along with roughening of the HK-2 membrane (Figure 1C). Next, we used flow cytometric analysis to investigate whether Pb^2+^ could enhance erythrophagocytosis by HK-2 cells. The extent of phagocytosis, as determined by the number of HK-2 cells positive for the erythrocyte marker, was significantly increased by Pb^2+^ exposure (*p* < 0.01; Figure 1D).

**Figure 2:**
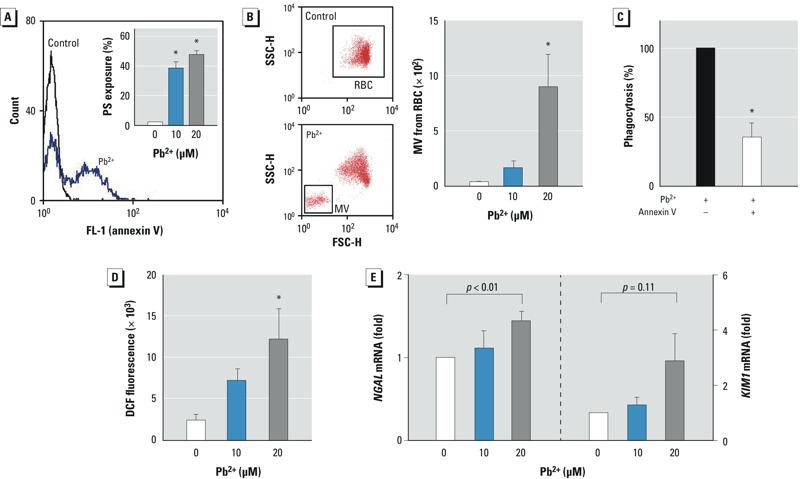
Role of Pb^2+^-induced erythrophagocytosis in renal tubular damage. (*A,B*) Erythrocytes (red blood cells; RBCs) were incubated with Pb^2+^ for 1 hr at 37°C, and flow cytometric analysis was used to determine externalization of phosphatidylserine (PS) in the outer membrane (*A*) or generation of microvesicles (MVs) from erythrocytes (*B*); representative histograms and quantified bar graphs are shown. (*C*) Pb^2+^-induced erythrophagocytosis in HK-2 cells was reversed by the addition of purified annex V, which masked externalized PS in erythrocytes. (*D,E*) Renal tubular damage determined after co-incubation of HK-2 cells with Pb^2+^-treated erythrocytes for 4 hr at 37°C. Generation of ROS was measured by dichlorofluorescein fluorescence in HK-2 cells by flow cytometry (*D*), and mRNA levels of the nephrotoxic biomarkers *NGAL* and *KIM1* were detected in HK-2 cells by qRT-PCR (*E*). Data were analyzed by one-way ANOVA followed by Duncan’s multiple range test (*A*,*B*,*D*) or Student’s *t*-test (*C*,*E*) to determine statistical significance. Values shown are the mean ± SE of more than three independent experiments. *p < 0.05 compared with the corresponding control.

*Role of Pb^2+^-induced erythrophagocytosis in renal tubular damage*. Previous studies have shown that erythrophagocytosis by macrophages is mediated by PS on the outer membrane of erythrocytes (Jang et al. 2011; Noh et al. 2010). To clarify whether Pb^2+^-induced PS externalization may contribute to erythrophagocytosis by renal tubular cells, we examined PS externalization in erythrocytes after Pb^2+^ treatment. The binding of annexin V, a marker for exposed PS, was increased in Pb^2+^-treated erythrocytes (Figure 2A), and the generation of PS-bearing MVs was also enhanced by Pb^2+^ (Figure 2B). Notably, when the exposed PS was neutralized by the added annexin V, Pb^2+^-induced erythrophagocytosis was significantly inhibited, suggesting that PS externalized on erythrocytes plays a key role in erythrophagocytosis by renal tubular cells (Figure 2C).

Next, we evaluated the potential role of erythrophagocytosis in renal tubular damage. Considering that erythrocytes contain a high amount of iron that can accumulate and induce excessive oxidative stress (Zager et al. 2002), we measured the generation of ROS in HK-2 cells. Co-incubation of Pb^2+^-treated erythrocytes increased ROS generation in HK-2 cells, indicating that Pb^2+^-induced erythrophagocytosis resulted in oxidative stress (Figure 2D). We also evaluated tubular damage by measuring mRNA levels of the representative nephrotoxicity biomarkers NGAL and KIM-1 (Figure 2E). *NGAL* mRNA expression in HK-2 cells was significantly increased by Pb^2+^-enhanced erythrophagocytosis, and there was a trend of increased KIM-1 expression although statistical significance was not achieved (*p* = 0.11).

**Figure 3:**
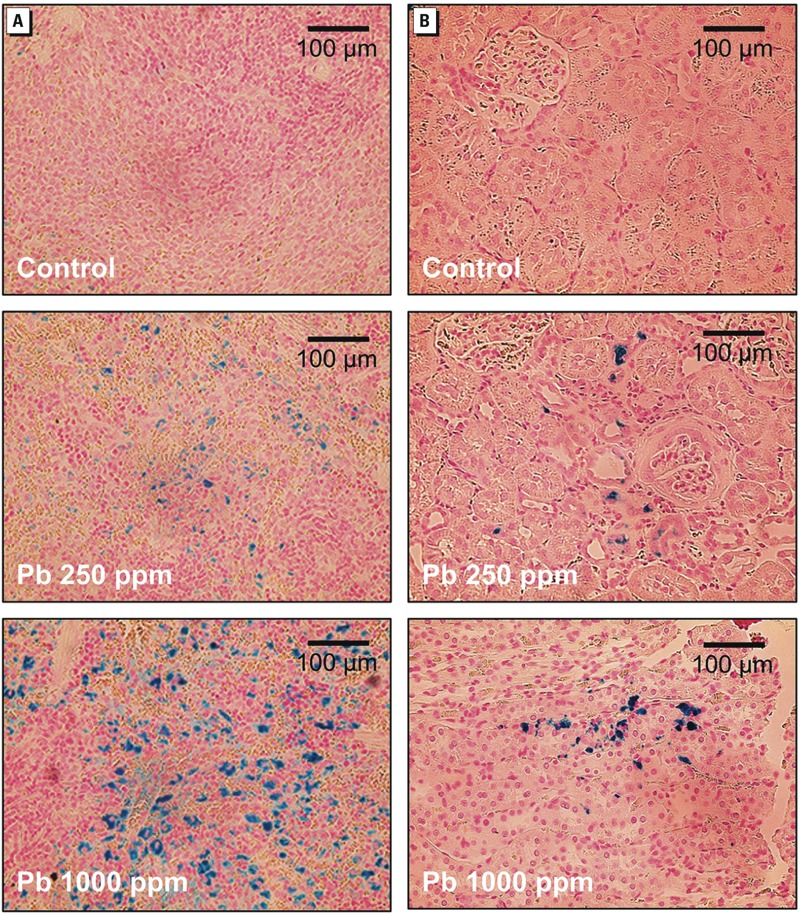
Representative photomicrographs showing the accumulation of iron in the spleen (*A*) and kidney (*B*) from rats treated with Pb^2+^ in drinking water [0 (control), 250, or 1,000 ppm] for 12 weeks*.* Iron is indicated by Prussian blue staining; bars = 100 μm.

**Figure 4:**
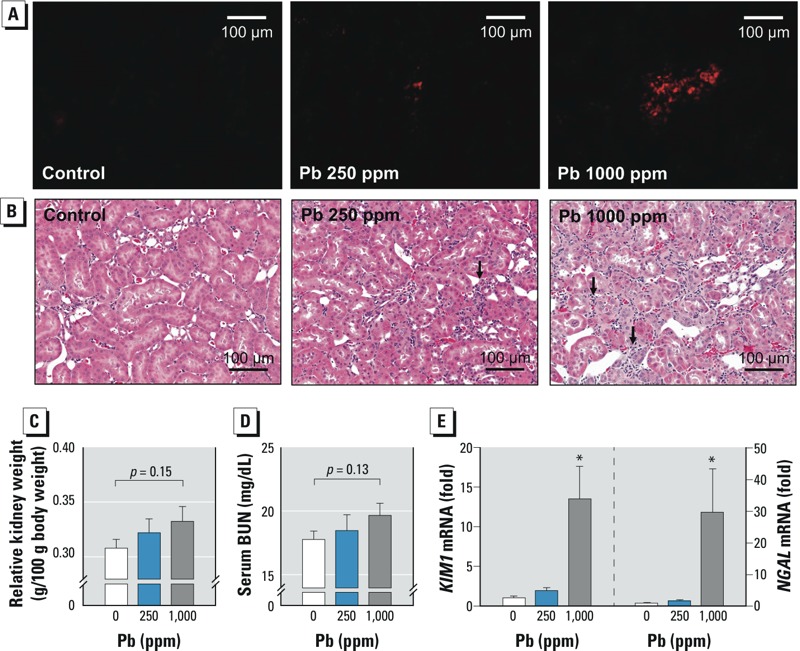
Renal damage associated with erythrophagocytosis in rats treated with Pb^2+^ [0 (control), 250, or 1,000 ppm] for 12 weeks. (*A*) Generation of ROS in kidney measured by DHE fluorescence. (*B*) Representative photomicrographs showing histopathological changes evaluated by H&E staining; bars = 100 μm. Relative kidney weight (*C*) and the level of BUN in serum (*D*). (*E*) Changes in *NGAL* and *KIM1* mRNA levels in kidney detected by qRT-PCR. Values are the mean ± SE of 6–9 rats/group; data were subjected to one-way ANOVA followed by Student’s *t*-test (*C,D*) or Duncan’s multiple range test (*E*). *p < 0.05 compared with the corresponding control.

In vivo *erythrophagocytosis in the kidney.* To evaluate the relevancy of our findings, we investigated the contribution of erythrophagocytosis in Pb^2+^-associated nephrotoxicity *in vivo*. After exposure of rats to Pb^2+^ (0, 250, or 1,000 ppm) in drinking water for 12 weeks kidney, spleen, and blood were isolated for biochemical and histological analysis. BLLs were 2.11 ± 0.54 μM for the 250-ppm Pb^2+^ group and 3.53 ± 0.84 μM for the 1,000-ppm Pb^2+^ group; BLLs of control rats were below the detection limit. The lead levels in kidney samples were 0.08 ± 0.05 μg/g, 12.29 ± 4.63 μg/g, and 26.67 ± 3.67 μg/g for the 0-, 250-, and 1,000-ppm Pb^2+^–treated rats, respectively. In Pb^2+^-treated rats, we observed iron accumulation in the spleen (Figure 3A), which was in agreement with our previous report (Jang et al. 2011), and in the kidney (Figure 3B). In addition, ROS generation was observed in kidney tissue from Pb^2+^-treated rats (Figure 4A).

*Evaluation of renal damage associated with Pb^2+^ treatment.* Along with iron accumulation and ROS generation in the kidney, we sought to determine whether Pb^2+^ treatment induced kidney damage. Histopathological examination revealed Pb^2+^-induced morphological alterations, such as tubulointerstitial lesions (characterized by basophilic regenerating tubules with altered morphology of epithelial cells) and interstitial lymphotic cell infiltration (Figure 4B). Conventional nephrotoxicity markers, such as relative kidney weight and serum BUN, were increased in kidneys from Pb^2+^-treated rats, but values were not statistically significant (Figure 4C,D). In contrast, mRNA levels of the nephrotoxic biomarkers (KIM-1 and NGAL) were significantly increased in kidneys from Pb^2+^-treated rats (Figure 4E), supporting Pb^2+^-induced kidney damage.

**Figure 5:**
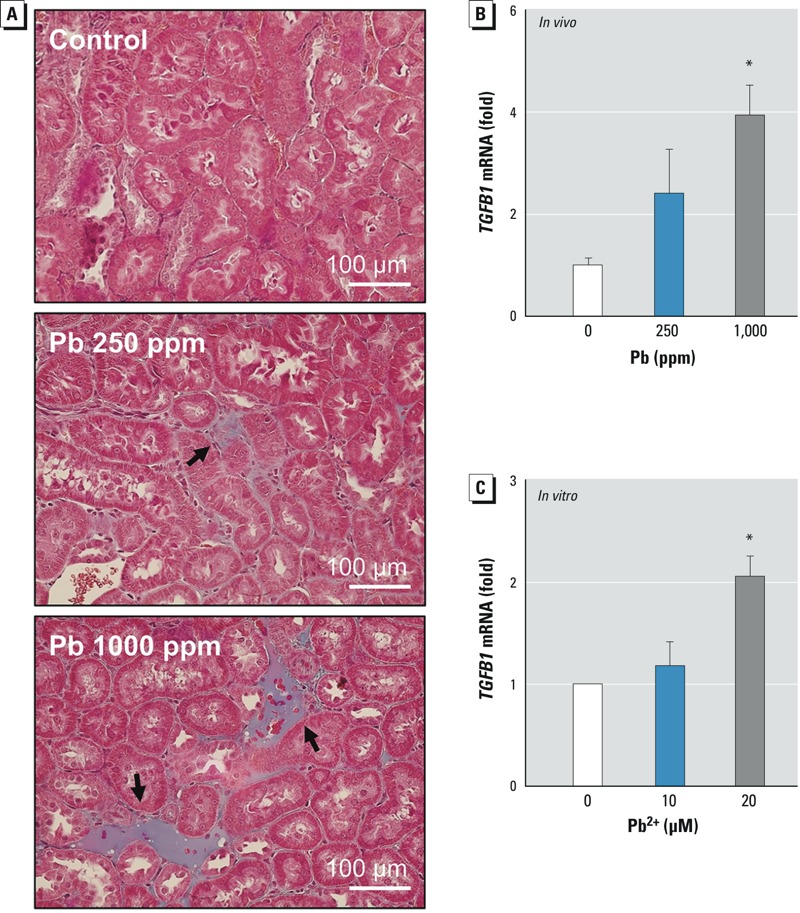
Renal fibrosis following Pb^2+^ exposure. (*A,B*) Rats were treated with Pb^2+^ [0 (control), 250, or 1,000 ppm] for 12 weeks, and kidneys were isolated. (*A*) Representative photomicrographs showing fibrotic collagen deposition examined by Masson trichrome staining (black arrows); bars = 100 μm. (*B*) The level of *TGFB1* mRNA in the kidney quantified by qRT-PCR; values shown are the mean ± SE of 5 rats/treatment group. (*C*) mRNA level of TGF-β in HK-2 cells after co-incubation with Pb^2+^-treated erythrocytes [0 (control), 10, or 20 μM Pb^2+^] for 4 hr; values shown are the mean ± SE of five experiments. Data were subjected to one-way ANOVA followed by Duncan’s multiple range test. *p < 0.05 compared with control.

Chronic exposure to Pb^2+^ has been associated with renal fibrosis (Cramér et al. 1974), which could ultimately disrupt normal kidney function. To evaluate the role of erythrophagocytosis in Pb^2+^-associated renal fibrosis, we examined collagen accumulation and the induction of TGF-β, an important mediator for renal fibrosis. We observed increased collagen deposition and increased *TGFB1* mRNA expression in the kidneys of rats treated with Pb^2+^ (Figure 5A,B). Notably, the expression of TGF-β was also significantly increased in HK-2 cells co-incubated with Pb^2+^-treated erythrocytes (Figure 5C), demonstrating that Pb^2+^-associated erythrophagocytosis may play important roles in Pb^2+^-associated renal fibrosis.

## Discussion

In the present study, we found that erythrocytes may play an important role in the potentiation of lead (Pb^2+^)-induced nephrotoxicity through PS-mediated erythrophagocytosis. Although treatment with Pb^2+^ alone (up to 20 μM) failed to induce statistically significant cytotoxicity in HK-2 renal tubular cells, co-incubation of HK-2 cells with Pb^2+^-treated erythrocytes (at a concentration as low as 10 μM) significantly decreased cell viability. Increased oxidative stress and up-regulation of nephrotoxic biomarkers, such as NGAL, were also observed in HK-2 cells co-incubated with Pb^2+^-treated erythrocytes. Moreover, TGF-β, a marker of fibrosis, was significantly up-regulated. In addition, the role of erythrocytes in lead-induced nephrotoxicity was also observed *in vivo* in rats treated with Pb^2+^ via drinking water for 12 weeks, as shown by increased iron deposition and generation of oxidative stress in renal tissues, as well as increased fibrosis and expression of KIM-1, NGAL, and TGF-β.

Lead contamination is a significant environmental threat to public health. Among the lead-associated adverse health effects, nephrotoxicity, represented by proximal tubular nephropathy and interstitial fibrosis, has been frequently reported in epidemiological studies (Cramér et al. 1974; Navas-Acien et al. 2009). High BLL (70–80 μg/dL) is known to be an established risk factor for chronic renal damage (Ekong et al. 2006; Fadrowski et al. 2010). Despite robust epidemiological evidence, the mechanisms underlying lead-induced nephrotoxicity have not been fully understood. *In vitro* studies employing renal cells revealed that kidney cells were somewhat resistant to lead-induced cytotoxicity—as indicated by our results—and that higher concentrations of lead than the reported human BLLs were required to manifest oxidative stress and cytotoxicity (Stacchiotti et al. 2009). In our study, we observed that erythrophagocytosis by kidney cells may underlie lead-induced nephrotoxicity and cytotoxicity, providing an important clue to explain the discrepancy. Although we cannot completely exclude the possibility of direct effects of lead on the kidney after chronic exposure *in vivo*, our data strongly suggest that erythrophagocytosis and subsequent iron deposition could contribute to lead-associated kidney damage. Experiments are needed to determine whether blocking or neutralizing the externalization of PS or the attenuation of iron-mediated ROS generation could reverse lead-associated nephrotoxicity.

Heavy metals that are absorbed into the bloodstream are continuously exposed to and accumulated into erythrocytes. In the case of lead, 99% of blood lead is associated with erythrocytes (Hernández-Avila et al. 1998; Schütz et al. 1996). Lead induced disruption of membrane-lipid asymmetry and subsequent PS externalization in erythrocytes, which is mediated by alteration of aminophospholipid translocase activities (Jang et al. 2011; Kempe et al. 2005) and activation of transbilayer lipid movement (Shettihalli and Gummadi 2013). Externalization of PS by lead treatment can induce erythrophagocytosis by interacting with scavenger receptors on macrophages in the spleen (Willekens et al. 2005). PS-externalized erythrocytes can be also engulfed by other tissue, which can eventually cause certain pathogenic effects (Fens et al. 2012; Otogawa et al. 2007). Otogawa et al. (2007) reported that a high-fat diet induced PS externalization on erythrocytes and erythrophagocytosis by Kupffer cells in the liver, resulting in inflammation and hepatic fibrosis. Fens et al. (2012) demonstrated that activated endothelial cells were capable of erythrophagocytosis, leading to endothelial cytotoxicity. Ichimura et al. (2008) showed that KIM-1 in kidney tubular epithelial cells specifically recognized PS, and was responsible for phagocytosis. Loss of phospholipid asymmetry in erythrocytes was also observed in uremia (Kong et al. 2001) and chronic renal failure (Bonomini et al. 1999, 2001). Although erythrophagocytosis by renal tubular cells has been confirmed in several studies (Madsen et al. 1982; Mimura et al. 2008), its pathophysiological significance has not been elucidated. The results of the present study indicate that lead-induced erythrophagocytosis was associated with increased oxidative stress and histological changes in renal cells. Additional research is needed to examine the potential role that erythrophagocytosis may play in the pathogenesis of kidney disease.

In addition to lead accumulated in erythrocytes being transported to the kidney, the pathological role of erythrophagocytosis may stem from iron abundance in erythrocytes. Erythrocytes contain about 70% of the body’s iron content in the form of hemoglobin, and abnormal uptake of damaged erythrocytes by intact tissues can result in iron accumulation and subsequent cellular damage. Iron overload stimulates ROS generation by an oxidation–reduction reaction (Fraga and Oteiza 2002; Valko et al. 2005). Deposition of large amounts of free iron is known to cause critical damage in the liver, heart, and other organs (Rasmussen et al. 2001). In the kidney, ROS-mediated lipid peroxidation (Zager et al. 2002) and renal fibrosis (Kovtunovych et al. 2010) can be induced by iron accumulation, ultimately leading to tubular cytotoxicity (Zager et al. 2004). The present study suggests that erythrophagocytosis may explain the potential source of iron accumulation and ROS generation in the kidneys following lead exposure.

According to the CDC (1997), the average BLL in adults is 1–2 μg/dL (~ 0.05–0.1 μM), and BLLs > 10 μg/dL (0.48 μM) are defined as lead poisoning. Lead exposure–associated nephrotoxicity has been observed at BLLs as low as 5 μg/dL (Ekong et al. 2006), and BLL is a known risk factor for nephropathy (Muntner et al. 2003). During 2002–2011, the Adult Blood Lead Epidemiology and Surveillance program identified 11,536 adults in the United States with very high BLLs (≥ 40 μg/dL), and 19% of these adults had these very high BLLs during ≥ 2 calendar years (CDC 2013), showing that lead exposures continue to occur at unacceptable levels. In rat models, histological and functional damages in kidney became evident at BLLs of 36–72 μg/dL (Liu et al. 2012a; Mahaffey et al. 1980). To achieve high BLLs for the experiments on the mechanism underlying lead-associated nephrotoxicity, we treated rats with 1,000 ppm Pb^2+^ in drinking water; in lead-treated rats, the mean BLL was 73.5 ± 17.4 μg/dL and the mean lead levels in kidney tissue was 26.67 ± 3.67 μg/g. Although the achieved BLL was epidemiologically reasonable, the lead level in drinking water may appear rather high; however, considering that 80% of environmental lead exposure is from other sources, such as food or inhalation, we consider the adoption of these high lead levels in drinking water necessary in our experimental system because drinking water was the only source of lead exposure.

In our *in vitro* experiments, we observed that co-incubation of HK-2 cells with Pb^2+^-treated erythrocytes (concentrations up to 20 μM for 1 hr) resulted in erythrophagocytosis and subsequent cytotoxicity. Although we could not extend the duration of Pb^2+^ exposure beyond 1 hr in our experimental setting due to technical limitations in maintaining erythrocyte integrity for 24-hr co-incubation, we believe that prolonged exposure to lower concentrations of Pb^2+^ could affect renal tubular viability through PS-exposure–mediated erythrophagocytosis. In a previous study (Jang et al. 2011), we demonstrated that the level of PS externalization obtained using Pb^2+^ 20 μM for 1 hr was similar to that obtained using Pb^2+^ 0.5 μM for 24 hr, suggesting that erythrophagocytosis can be induced at a much lower concentration of Pb^2+^ when exposed chronically, as observed in a real-life scenario.

We observed that erythrocyte uptake in the kidney induced iron deposition, ROS generation, and renal fibrosis. Fibrosis is a major determinant of progressive renal damage leading to end-stage renal failure (Eddy 1996), and it has been frequently observed in a lead-exposed population (ATSDR 2007). In the present study, we found typical characteristics of tubulointerstitial fibrosis, such as tubular loss and accumulation of collagen, the most abundant of the extracellular matrix (ECM) proteins in kidneys from lead-exposed rats. ECM is primarily produced by myofibroblasts (LeBleu et al. 2013); however, the active role of tubular epithelial cells in fibrosis has also been reported. In pathological states such as diabetes and hypertension, the potent profibrotic cytokine TGF-β is produced by tubular cells (Isaka et al. 2000), and stimulates renal fibroblasts to produce ECM (Vallon and Thomson 2012; Zhao et al. 2008). TGF-β also induces fibrogenic transdifferentiation of tubular epithelial cells to harbor ECM-producing character (Li et al. 2002). TGF-β expression in tubular cells could be induced by excessive oxidative stress (Vallon and Thomson 2012; Zhao et al. 2008). In the present study, we observed ROS generation and TGF-β up-regulation in the kidney of lead-exposed rats *in vivo* and tubular cells undergoing erythrophagocytosis *in vitro*, suggesting the potential contribution of lead-induced erythrophagocytosis to renal fibrosis. These findings are in agreement with a previous study that showed the up-regulation of NGAL and KIM-1 in tubular cells to be key renal injury biomarkers (Lock 2010).

## Conclusion

The results of the present study suggest that lead exposure can lead to the externalization of PS and generation of MVs in erythrocytes, which appears to be associated with increased erythrophagocytosis by renal tubular cells. Our data support the hypothesis that erythrophagocytosis seems to be associated with increased ROS generation, induction of nephrotoxicity biomarkers, TGF-β up-regulation, and decreased cell viability of renal tubular cells. Our *in vivo* experiments confirmed that chronic exposure to lead increased iron deposition in the kidney. The role that iron plays in lead-mediated oxidative stress and renal fibrosis warrants further research. We believe that our study gives a new insight into the mechanisms of lead exposure–associated nephrotoxicity.
